# Recycle, repair, recover: the role of autophagy in modulating skeletal muscle repair and post-exercise recovery

**DOI:** 10.1042/BSR20240137

**Published:** 2025-01-22

**Authors:** Jordan Acheson, Sophie Joanisse, Craig Sale, Nathan Hodson

**Affiliations:** 1 Department of Sport and Exercise Sciences, Manchester Metropolitan University, Institute of Sport, Manchester, U.K.; 2 School of Life Sciences, Queen’s Medical Centre, University of Nottingham, Nottingham, U.K.; 3 Faculty of Kinesiology and Physical Education, University of Toronto, Toronto, Ontario, Canada

**Keywords:** autophagy, skeletal muscle, recovery, exercise

## Abstract

Skeletal muscle is a highly plastic tissue that can adapt relatively rapidly to a range of stimuli. In response to novel mechanical loading, e.g. unaccustomed resistance exercise, myofibers are disrupted and undergo a period of ultrastructural remodeling to regain full physiological function, normally within 7 days. The mechanisms that underpin this remodeling are believed to be a combination of cellular processes including ubiquitin-proteasome/calpain-mediated degradation, immune cell infiltration, and satellite cell proliferation/differentiation. A relatively understudied system that has the potential to be a significant contributing mechanism to repair and recovery is the autophagolysosomal system, an intracellular process that degrades damaged and redundant cellular components to provide constituent metabolites for the resynthesis of new organelles and cellular structures. This review summarizes our current understanding of the autophagolysosomal system in the context of skeletal muscle repair and recovery. In addition, we also provide hypothetical models of how this system may interact with other processes involved in skeletal muscle remodeling and provide avenues for future research to improve our understanding of autophagy in human skeletal muscle.

## Introduction

During recovery from resistance exercise (RE), mechanically perturbed myofibers undergo rapid ultrastructural remodeling to regain full physiological function normally within a week [1,2]. Successive bouts of RE accustoms skeletal muscle to loading [3,4] and significant myofibrillar protein accrual (i.e. fiber cross-sectional area growth) can be observed after 10–12 weeks of resistance training (RT) [5–7]. This hypertrophic response is fundamentally driven by the combined effect of incremental mechanical loading and consistent dietary amino acid availability, which increases muscle protein synthesis (MPS) beyond muscle protein breakdown (MPB) for positive net protein balance over time [8,9]. The initial protein synthetic increase during early-stage RT is, however, likely indicative of a global stress response to novel exercise-induced muscle damage (EIMD) rather than hypertrophy adaptation *per se* [6,10–12]. While the necessity of this initial damage response for muscle hypertrophy has been debated [5,13,14], unaccustomed eccentric exercise evokes a degree of ultrastructural deformation and functional impairment [1], which likely needs to be attenuated for adaptive remodeling to ensue [6]. Therefore, identifying the mechanisms underpinning skeletal muscle recovery may uncover potential methods to enhance athletic performance or expedite training-induced adaptations.

While most post-exercise recovery strategies favor the stimulation of skeletal muscle anabolism, recent evidence suggests that changes in myofibrillar MPS do not directly explain improved exercise recovery when dietary protein is sufficient [15]. Further, an inability to synthesize myofibrillar proteins does not seem to be the underlying cause of delayed skeletal muscle recovery in aging rodents [16]. These intriguing data could imply that, in the context of muscle damage, MPB could be a modulating factor. However, despite the increasing need to consider proteostatic mechanisms holistically [16–18], our current understanding of human skeletal muscle catabolism is poor relative to anabolism [19]. Intracellular degradation is predominantly regulated by the calpain, ubiquitin-proteasome (UPS), and autophagolysosomal systems, each of which has their own distinct underpinning signaling pathways. Throughout this review, we will focus on the role of the autophagolysosomal system in skeletal muscle, with particular emphasis on its (potential) role during recovery from EIMD. For an in-depth overview of the role of the calpain and UPS systems in skeletal muscle, readers are directed to the review provided by Goll et al. [20].

## Overview of autophagy

Autophagy, meaning ‘self-eating’, is a conserved intracellular process originally conceptualized in 1963 by the discoverer of the lysosome, De Duve [21], and mechanistically expanded upon in yeast by Ohsumi [22,23] during the 1990s. Today, it is evident that autophagy is a pivotal quality control mechanism through which mammalian cells maintain tissue homeostasis under basal conditions [24,25] and in response to physiological stress [25,26]. Unlike the protein-specific UPS, autophagy also facilitates the degradation of lipids, carbohydrates, and nucleotides via acid hydrolysis. An energy-depleted and/or metabolically perturbed intracellular environment initiates the autophagic process, allowing redundant structures to be recycled into their constituent metabolites for anabolic (i.e. growth of cellular components) or catabolic (i.e. adenosine triphosphate resynthesis) repurposing. Abnormal clearance of such components can be detrimental to skeletal muscle health; thus, it is unsurprising aberrant autophagy has been linked to a variety of muscular diseases, the primary category being lysosomal storage disorders [27].

There are three known types of autophagy distinguishable by the method of cargo delivery to the lysosome: micro-autophagy, chaperone-mediated autophagy, and macro-autophagy. Although likely interconnected [28], a holistic discussion of these pathways is beyond the scope of this review; therefore, readers are referred elsewhere for overviews of chaperone-mediated [29] and micro-autophagy [30]. Macro-autophagy, henceforth referred to as autophagy, involves the intricate and highly coordinated interaction of autophagolysosomal machinery. Here, damaged or redundant organelles/cytosolic constituents are engulfed by nascent double-membrane autophagosome vesicles, which, in turn, fuse with lysosomes where the isolated cargo is catabolized. Originally thought to be an entirely non-selective process, more than 30 selective autophagy receptors have now been discovered [31] displaying how this pathway acts not only as a global stress response but can also target specific intracellular components.

## Molecular mechanism of autophagosome biogenesis and degradation

The autophagic process is complex but can be broken down into four distinct stages: (1) induction and nucleation of the pre-autophagosome phagophore, (2) expansion of the phagophore membrane, (3) autophagosome maturation/lysosome fusion, and (4) hydrolytic degradation and efflux of metabolites.

**Figure 1:**
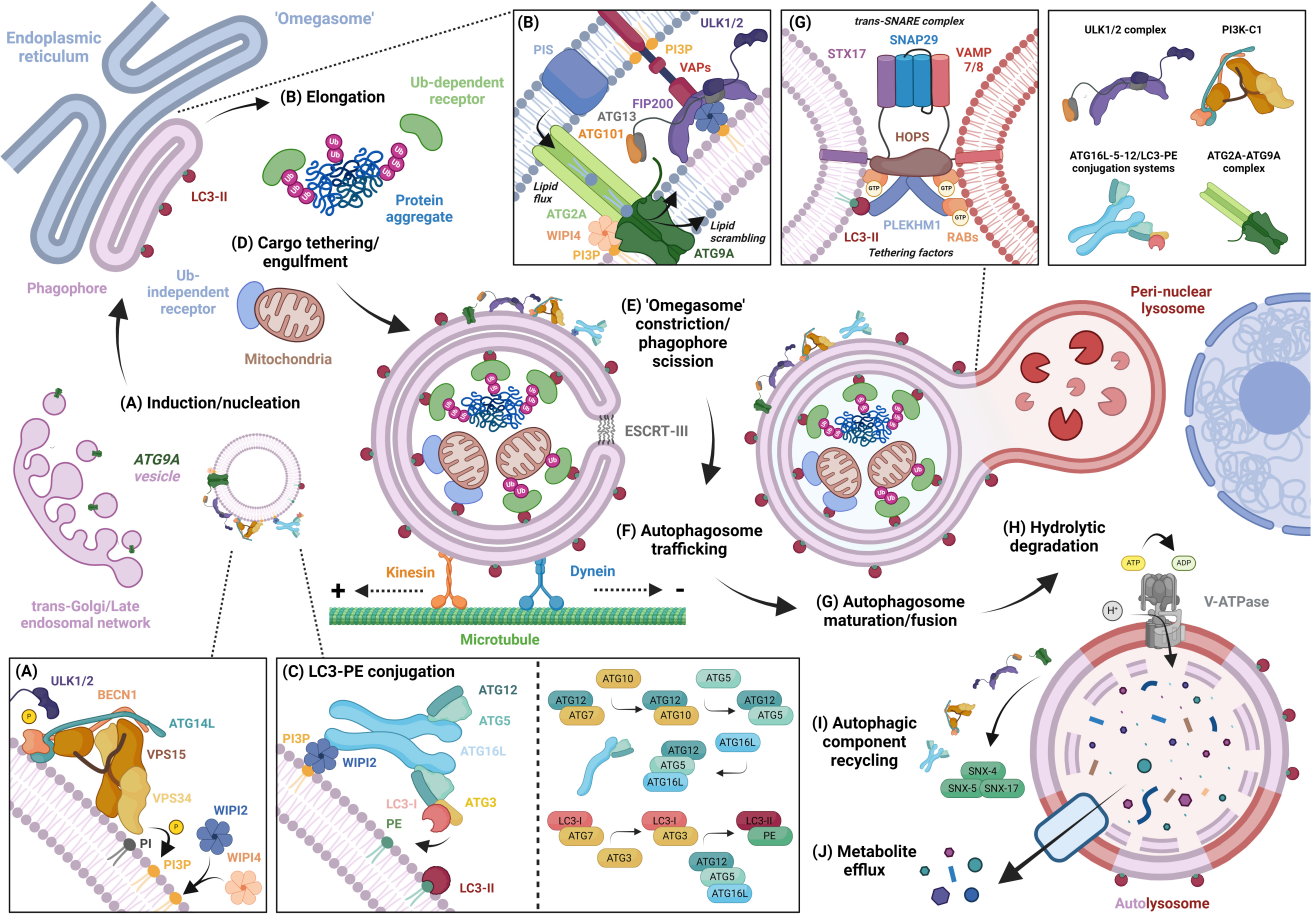
General mechanism of autophagosome biogenesis and degradation. (**A**) Activated ULK1/2 complexes localis ze at the phagophore induction site and phosphorylates Beclin-1, allowing VPS34 to convert PI to PI3P for recruitment of WIPI proteins. (**B**) Recently nucleated phagophores are tethered to the ‘Omegasome’ by interactions between integral ER VAPs, ULK1/FIP200, and WIPI2. ATG2A associates with ATG9A via WIPI4 and transfers synthesis zed ER lipids to the elongating phagophore, which are equilibrated by the ATG9A lipid scramblase. (**C**) LC3/GABARAP proteins are embedded into the autophagic membrane through a series of ubiquitin-like reactions mediated by ATG7 and ATG3 as well as the ATG12-5-16L complex. (**D**) Autophagic cargo is tethered to LC3-II on the nascent phagophore membrane through connecting autophagy receptors. (**E**) Fully elongated phagophores are sealed by ESCRT machinery and disassociate from their ER donor through Omegasome constriction. (**F**) Newly formed autophagosomes are transported towards along microtubules by kinesin or dynein motor proteins, where they eventually encounter lysosomes. (**G**) GTP-loaded Rab proteins and LC3-II promote the tethering of the lysosomal and outer autophagosome membrane through PLEKHM1 and its HOPS complex effector allowing SNARE-mediated fusion of the membrane structures. (**H**) The V-ATPase acidifies the autolysosome lumen allowing hydrolysis of the inner autophagosome membrane, connecting autophagy adapto ors, and sequestered cargo. (**I**) Autophagic components are extracted from the autolysosome membrane by the SNX4-5-17 recycler complex and degraded materials are released into the cytosol (**J**). Created in BioRender. Acheson, J. (2024) BioRender.com/a02s604.

Autophagic induction and phagophore nucleation are primarily coordinated by the Unc51-like kinase 1/2 (ULK1/2) autophagy initiation complex, comprising the ULK1/2 kinase, autophagy-related gene (ATG) 13, ATG101, and scaffold protein focal adhesion kinase family interacting protein of 200 kD (FIP200), and the autophagy specific class III phosphatidylinositol 3-kinase complex 1 (PI3K-C1), containing the phosphatidylinositol 3-phosphate (PI3P) kinase vacuolar protein sorting (VPS) 34, VPS15, ATG14L, and Beclin-1. Upon activation, ULK1/2 complexes are recruited to the phagophore initiation site where they exert kinase activity toward PI3K-C1 for PI3P production (Figure 1A) [32,33]. Localized accumulation of PI3P at the initiation site recruits WD repeat domain (WIPI) PI3P-binding proteins, triggering the assembly of downstream autophagy machinery for membrane elongation [34].

Mammalian autophagosomes are predominantly formed in association with an endoplasmic reticulum (ER) subdomain with an Ω-like shape, fittingly termed the ‘omegasome’ [35]. Here, phagophore-ER contact sites are established between integrated ER VAMP-associated proteins (VAPs) and FIP200/ULK1 in a PI3P-dependent manner [36], while ATG2A and ATG9A transfer lipids from the ER to the elongating phagophore membrane (Figure 1b) [37,38]. ATG9A is trafficked to the phagophore assembly site within Golgi/endosome-derived vesicles [39,40], which form the initial phagophore seed [41] and begin to accumulate autophagic machinery [33]. The ubiquitin-like ATG16L-ATG5-ATG12 conjugation system associates with the growing phagophore to convert cytoplasmic microtubule-associated protein 1A/1B-light chain 3 (LC3-I) into membrane-bound phosphatidylethanolamine (PE) conjugated LC3-II [34,42–45] in a series of reactions involving ATG10, ATG7, and ATG3 (Figure 1C) [46–49].

LC3 isoforms and their gamma-aminobutyric acid receptor-associated protein (GABARAP) subfamily are structurally like other ubiquitin-like proteins but contain two extra α-helices that act as docking regions for autophagy-related proteins [50]. Several ATGs within the ULK1/2 complex, PI3K-C1, and ATG2A lipid-transferase harbor LC3-interacting regions; thus, it is thought that lipidated LC3 and GABARAP accelerate phagophore expansion by providing additional scaffolding sites for autophagic machinery [51–53]. Furthermore, during selective autophagy, LC3/GABARAP mediates tethering of predetermined cargo to the inner phagophore membrane (Figure 1D). Ubiquitin-sensitive autophagy receptors, such as sequestosome-1 (p62), connect ubiquitinated cargo to PE-conjugated LC3/GABARAP through LC3 and ubiquitin-binding domains [54], whereas ubiquitin-independent receptors such as BCL2/Adenovirus E1B 19 kDa protein-interacting protein 3 (BNIP3) and BNIP3-like (BNIP3L/NIX), directly bind cargo to LC3/GABARAP [55,56].

Autophagosomes are formed once fully elongated phagophores undergo endosomal sorting complexes required for transport (ESCRT) mediated membrane scission [57–59] and omegasome constriction, allowing the autophagosome to dissociate from its membrane donor (Figure 1E) [60,61]. Newly formed autophagosomes are then bound to motor scaffold proteins and transported along the microtubule network via dynein [62–64] and kinesin [65] toward juxtanuclear lysosomes (Figure 1F) [66]. Here, concerted actions between Rab guanosine triphosphatases (GTPases), LC3 proteins, homotypic fusion and protein sorting (HOPS)-tethering factors, and soluble N-ethylmaleimide-sensitive factor attachment protein receptors (SNARE) promote tethering and fusion of the outer autophagosome and lysosomal membranes (Figure 1G), subsequently creating an autolysosome [67–70].

Autophagic catabolism within autolysosomes involves the hydrolysis of the inner autophagosome membrane, the cargo it surrounds, and the connecting autophagy adaptors. Such degradation can be carried out by over 60 lysosomal hydrolases [71], which require acidification of the autolysosome lumen by vacuolar H^+^-adenosine triphosphatases (v-ATPase) (Figure 1H). Importantly, however, recent evidence has suggested that proteins of the outer autophagosome membrane are recycled for future use (Figure 1I) [72,73]. Similarly, for efficient autophagy [74], lysosomal bodies must be extracted from the autolysosome through a separate process termed autophagic lysosome reformation (reviewed in-depth elsewhere [75]). Following catabolism within the autolysosome, constituent elements of degraded material are released into the cytosol through various transporters/channels for subsequent recycling into new cellular components (Figure 1J) [76].

The autophagolysosomal system is a highly regulated pathway and there are a myriad of stages that have the potential to be affected by stimuli such as mechanical disruption/damage of tissue. This is important as common markers of autophagosomes (e.g. LC3-II and p62) fluctuate depending on their rate of synthesis and autophagic degradation [77], meaning ‘static’ assessments of these proteins could misrepresent true rates of autophagy. Therefore, control comparators where autophagosome degradation has been inhibited (e.g. with colchicine) are required to confirm whether autophagy ‘flux’ (i.e. autophagosome synthesis and degradation) has increased or decreased in response to experimental conditions.

## Upstream regulation of autophagy

### Nutrient-sensitive autophagy signaling

Our understanding of the upstream signals that regulate autophagy primarily stems from *in vitro* or animal studies utilizing nutrient withdrawal or pharmaceutical autophagy inhibitors. During periods where nutrients are replete, the active mammalian target of rapamycin complex 1 (mTORC1) inhibits the ULK1/2 complex by phosphorylating ULK1^ser757^ and ATG13^ser258^ [78–81]. Upstream of mTORC1, the growth factor/insulin-sensitive protein kinase B/AKT (AKT) phosphorylates transcription factor forkhead box O3 (FOXO3) at several inhibitory sites, promoting its interaction with cytosolic 14-3-3 to suppress autophagy gene transcription [82–85]. In a similar mechanism, both AKT and mTORC1 prevent coordinated lysosomal enhancement and regulation (CLEAR) gene network expression by phosphorylating members of the microphthalmia/transcription factor E (MiT/TFE) family, such as transcription factor EB (TFEB) and transcription Factor Binding to IGHM Enhancer 3 (TFE3) [86–88].

**Figure 2:**
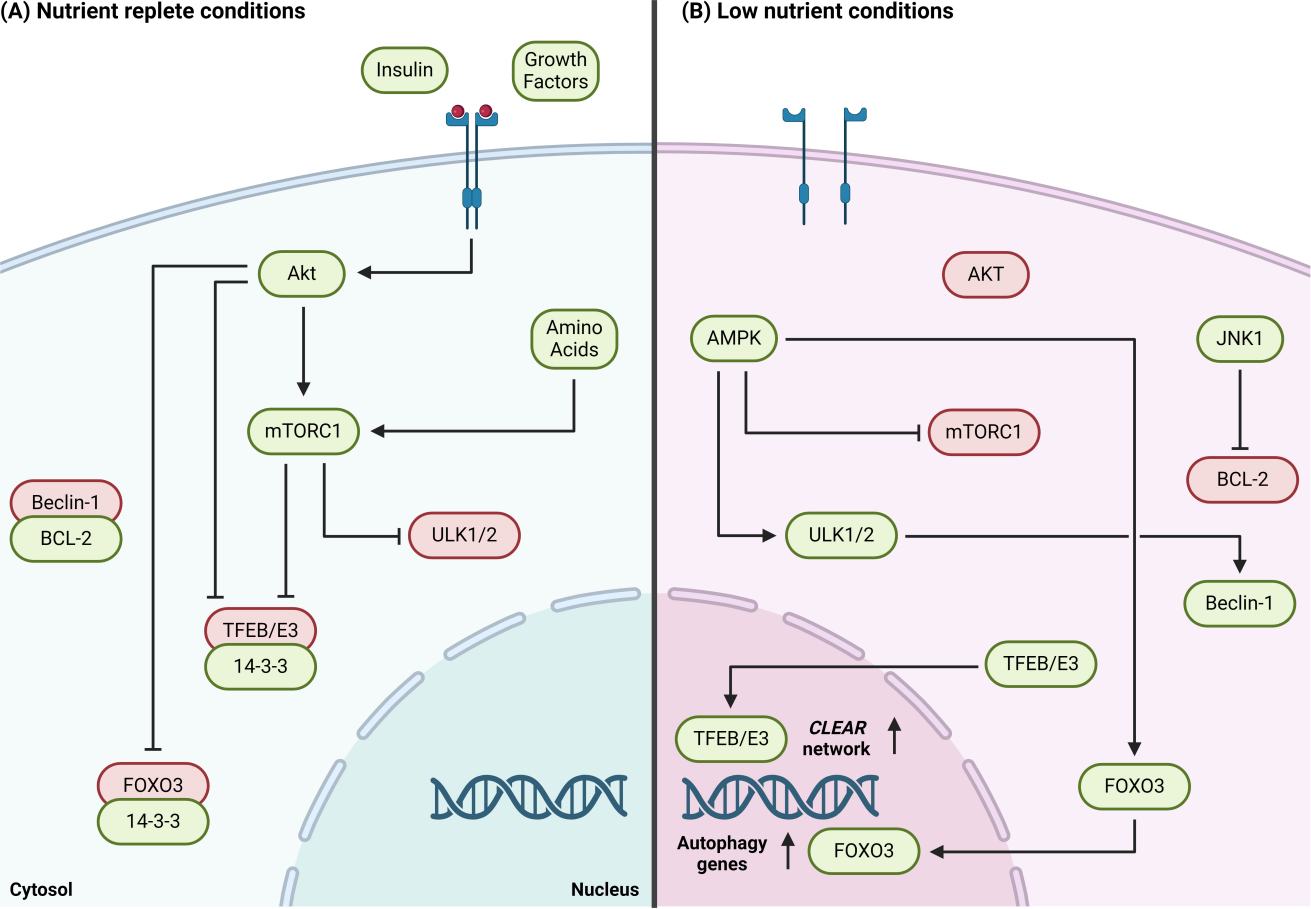
Upstream regulators of nutrient-sensitive autophagy. (**A**) During periods where growth factors and amino acids are replete, the AKT/mTORC1 pathway negatively regulates autophagy induction and transcriptional programmes. mTORC1 directly phosphorylates and inhibits ULK1/2, ULK1/2, and ATG13. TFEB/E3 are phosphorylated by both AKT and mTORC1 and sequestered in the cytosol by 14-3-3 proteins, whilst e AKT similarly phosphorylates and prevents FOXO3-mediated autophagy gene expression. Autophagosome biogenesis is suppressed through BCL-2 -mediated inhibition of Beclin-1. (**B**) In low nutrient conditions, a reduction in AKT/mTORC1 signalling alleviates the negative inhibition of the ULK1/2 complex. The energy- sensor AMPK simultaneously phosphorylates and activates ULK1/2, allowing it to phosphorylate Beclin-1, which has been released forom BCL-2 by JNK1. FOXO3 and TFEB/E3 translocate to the nucleus and upregulate autophagy and CLEAR network gene expression due to the reduction in AKT/mTORC1 activity and direct phosphorylation of FOXO3 by AMPK. Created in BioRender. Acheson, J. (2024) BioRender.com/r89e937.

In contrast, during nutrient deficiency, a reduction in mTORC1 signaling alleviates the induction complex’s negative inhibition on ULK1/2 and TFEB, allowing autophosphorylation of ATG13 and FIP2000 by ULK1/2 [79,80] and TFEB nuclear translocation for CLEAR gene network expression [89]. Meanwhile, an increase in cellular adenosine monophosphate (AMP):triphosphate (ATP) activates the energy sensor 5′ AMP-activated protein kinase (AMPK), which promotes autophagy induction by phosphorylating ULK1/2 at multiple serine residues [90–92] and elevates autophagy-related gene expression via phosphorylation of FOXO3 [92,93]. AMPK may also indirectly promote autophagy, given its ability to inhibit mTORC1 through phosphorylation of tuberous sclerosis protein and raptor [78,90–92]. Finally, phosphorylation of B-cell leukemia/lymphoma 2 protein (BCL-2) at several residues by starvation-activated c-Jun N-terminal protein kinase 1 (JNK1) attenuates BCL-2’s negative inhibition of Beclin-1 [94,95]. These opposing mechanisms allow cellular autophagy to be regulated temporally in response to changes in nutrient status (Figure 2).

### Redox/calcium-sensitive autophagy signaling

**Figure 3:**
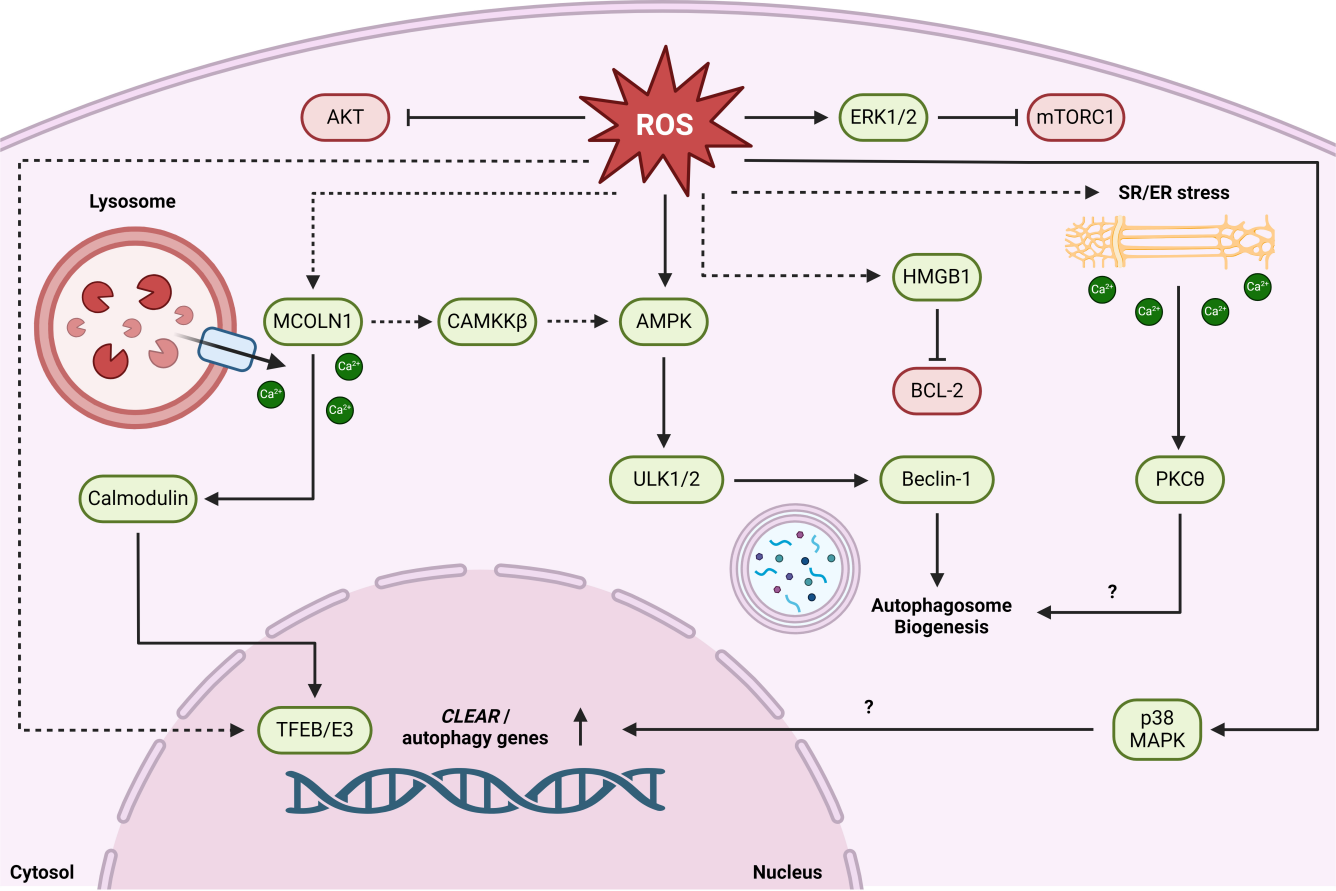
Redox and calcium-related autophagy signaling. Perturbations in cellular redox and intracellular calcium homeostasis can result in autophagy induction and related gene expression. ROS increase ULK1/2 complex activity through allosteric activation of AMPK and attenuation of AKT/mTORC1 activity. Lysosomal calcium efflux through MCOL1N, which can be induced by ROS, results in autophagy enhancement through CaMKKβ/AMPK activation and by activating TFEB via calmodulin. ROS may also promote autophagy gene expression through direct oxidation of TFEB/E3 and increasing p38 MAPK signalling. Oxidative stress results in sarcoplasmic reticulum efflux, associated with autophagosome biogenesis through activation of PKCθ. ROS promote HMGB1 relocation to the cytosol where it promotes autophagy induction by competitively binding BCL-2. Dashed lines represent autophagic pathways that remain to be documented in skeletal muscle. Created in BioRender. Acheson, J. (2024) BioRender.com/f77k718.

While there are various potential upstream autophagic regulators, disturbance of intracellular redox and calcium homeostasis has been strongly implicated in skeletal muscle autophagy (Figure 3) [96–98]. Mitochondrial-derived reactive oxygen species (ROS) are thought to be key regulators of contraction-induced autophagy induction [99–101]. Mechanistically, ROS activate AMPK by reducing cellular ATP [102], upregulate autophagy by attenuating AKT signaling [103,104], and directly oxidize MiT/TFE transcription factors to promote autophagolysosomal gene expression [105]. Furthermore, ROS stimulate lysosomal calcium release by oxidizing the lysosome calcium channel mucolipin-1/TRPML1 (MCOLN1) [106,107] which, in turn, promote TFEB nuclear translocation to elevate lysosomal/autophagy-related gene expression [108]. MCOLN1 also activates the AMPK-effector calcium/calmodulin-dependent protein kinase kinase β (CaMKKβ), resulting in phosphorylation of ULK1 and Beclin-1 [109].

Alike starvation-induced autophagy, exercise promotes the phosphorylation and dissociation of BCL-2 from Beclin-1, albeit via a differing mechanism [110]. In addition, exercise-induced phosphorylation of p38 mitogen-activated protein kinases (MAPK) has been implicated in upregulating autophagy gene expression [111], which could reflect upstream regulation by oxidative stress [112] or inflammatory receptor activation [113]. Cytosolic calcium mobilization in response to sarcoplasmic reticulum (SR) stress [114,115] may also promote autophagosome formation via protein kinase C theta (PKCθ) [116], while ROS produced during lactate clearance stimulates phosphorylation of extracellular signal-regulated kinases 1/2 (ERK1/2), thereby inhibiting mTORC1 and promoting autophagy flux [117]. Collectively, these mechanistic studies highlight potential redox and calcium-sensitive signaling cascades that may be upregulated during recovery from strenuous exercise, albeit the degree to which such pathways influence autophagy flux in human skeletal muscle is yet to be determined.

## Exercise-induced muscle damage

### Defining exercise-induced muscle damage

It is well known that mechanical and/or metabolic stress during strenuous physical exercise causes temporary muscle damage and functional impairment [2,118–121]. Human experimental models of EIMD clearly show that repeated isolated eccentric muscle contractions significantly disrupt myofiber integrity and muscle force generating capacity [122–129], the most appropriate proxy of ultrastructural damage [130,131]. In severe cases of EIMD, evidence of myofiber necrosis, such as intramyofiber immune cell infiltration [127,129,132,133], particularly in dystrophin-negative myofibers [133], is also observed. Such effects are often preceded by a substantial (≈50%) loss of muscle force generating capacity that requires > 7 days to recover [127,132,133]. In contrast, more traditional resistance and endurance-type exercise results in milder muscle strength losses and shorter recovery periods [134,135], although ultrastructural damage is noted following high-intensity RE [6,136–139] and downhill running [140,141], reflecting the eccentric demand of these modalities.

## Mechanisms of exercise-induced muscle damage

To understand how autophagy may contribute to skeletal muscle recovery, it is important to consider the etiology of EIMD. This can be divided into two distinct phases: the initial primary damage phase, relating to the immediate exercise-induced disruption of intracellular proteins and organelles, and the secondary damage phase, which occurs as an autogenic response to the initial damaging event.

### Primary damage

Eccentric loading of contractile proteins is likely the primary source of ultrastructural damage during weight-bearing exercise [142]. Compared to concentric loading, eccentric contractions recruit a lower number of motor units, which results in a greater amount of tensile stress per unit of muscle fiber area [143,144]. As a result, elongating sarcomeres progressively weaken and eventually stretch beyond myofilament overlap, subsequently placing a greater strain on surrounding structures (see Morgan’s [145] ‘popping sarcomere hypothesis’). In support of this theory, disruption of sarcomeres (e.g. Z-line/disk ‘smearing’ or myofilament disorganization), the t-tubule system, SR, and intermyofibrillar mitochondria can all be observed in skeletal muscle tissue immediately after eccentric exercise [146–148]. Furthermore, much of the acute post-exercise reduction in muscle function has been attributed to a rise in intracellular calcium concentrations and subsequent excitation-contraction uncoupling [148,149]. While the precise mechanism of cytosolic calcium influx is debated [150], evidence from both human and animal investigations suggests that stretch-mediated and/or oxidative disruption of t-tubules, sarcolemma, and the SR may be involved [122,148,151–153].

### Secondary damage

During the secondary phase, a rise in cytosolic calcium levels signals the activation of calcium-sensitive calpains [154–156]. It is thought that these non-lysosomal proteases disassemble damaged myofibrillar and cytoskeletal structures, subsequently allowing the UPS to degrade unbound protein fragments into smaller peptide chains [20]. Indeed, human eccentric exercise-induced myofibrillar disruption and loss of muscle function directly correlate with calpain activity [155], while proteasome activity increases during the post-exercise period [157]. However, given that the presence of myofibrillar disruption is often delayed [155,158,159], changes in tissue ultrastructure during the days following exercise likely reflect a remodeling response to strenuous loading rather than further ‘damage’. Elevated cytosolic calcium may also increase mitochondrial calcium uptake and ROS formation [160], which are known to cause lipid, protein, and DNA oxidation in exercised-human muscle [161]. However, recent animal evidence has shown that transient ROS formed by localized mitochondrial calcium uptake are important for sarcolemma repair and maintaining myofiber viability following eccentric-damage, whereas sustained increases in cellular ROS hinder homeostatic regain [162]. Other rodent-based studies suggest that redox imbalances created by eccentric exercise may promote mitochondrial calcium overload and permeability [160,163,164], which increases the risk of pro-apoptotic factors entering the cytosol [165].

Inflammation is another key factor associated with secondary EIMD. Various cytokines are elevated in skeletal muscle and the circulation following eccentric exercise (for an extensive list, see Paulsen et al. [1]), some of which have been shown to coordinate the immune and myogenic response in cultured human skeletal myotubes [166–169]. Neutrophils initially accumulate in damaged human skeletal muscle tissue with pro-inflammatory M1-like phagocytic macrophages predominating soon after [133,167]. These phagocytic cells contribute to muscle healing by clearing cellular debris and further regulating the immune response, although cell culture and rodent models of muscle injury indicate that neutrophils also exhibit a role in generating cytolytic intermediates during phagocytosis [170–174]. Mouse M1 macrophages differentiate into a pro-regenerative, anti-inflammatory M2 phenotype upon engulfing muscle debris *in vitro* [175], and this timely expression seems to be important for regulating satellite cell dynamics and healthy myofiber regeneration in both humans and mice [174–178]. Importantly, however, a distinct change in macrophage phenotype may not occur in human skeletal muscle during recovery from traditional, less damaging forms of RE, possibly reflecting a lack of need to degrade necrotic tissue [179]. Nevertheless, macrophage and satellite cell accretion remain tightly coupled in response to exercise training [180–182]; thus, interactions between immune and myogenic cells are likely important for muscle recovery regardless of myofiber necrosis.

### Autophagy is essential for rodent skeletal muscle health and regeneration

Studies of skeletal muscle-specific ATG-knockout (KO) rodents have uncovered the pivotal role autophagy plays in maintaining myofiber homeostasis. Maserio et al. [24] delineated the importance of basal autophagy by generating life-long and tamoxifen-inducible ATG7-KO mouse lines. These animals were unable to lipidate LC3 and had substantial LC3 and p62 build-up, indicating a blockage of autophagosome removal. Both genotypes displayed loss of force production, indices of muscle damage, and skeletal muscle atrophy, which coincided with increased proteolytic gene expression and diminished activity of protein translational machinery, suggestive of a catabolic phenotype. ATG5-KO also induces glycolytic myofiber atrophy, which is associated with autophagy protein build-up and impaired lysosome morphology within the intermyofibrillar space [183]. Intriguingly, slow-twitch muscle and measures of muscle fatiguability were observed to be unaffected by ATG5-KO, highlighting a need to consider muscle-fiber type when investigating the autophagic response.

Pare et al. [184] showed that mouse chronic ATG7-KO hindered muscle force-production and measures of contractility in both the fast-twitch dominant extensor digitorum longus (EDL) and slow-twitch soleus muscle, but these effects were more pronounced and had an earlier onset in EDL. Analyses of autophagy-sufficient muscle showed that the EDL had higher basal autophagic flux and both basal and starvation-induced LC3-II accumulation are negatively associated with skeletal muscle citrate synthase activity [185], indicating that muscle with low oxidative capacity has greater autophagic turnover. Notably, glycolytic muscle is more susceptible to MPS attenuation and subsequent muscle loss during catabolic conditions [186,187], including aging where the decline in fast-twitch muscle size and function has been associated with dysfunctional autophagy [188]. However, preferential glycolytic muscle fiber wasting in sarcopenic muscle appears to be mTORC1 independent whereby inflammatory cytokines suppress autophagy via FYN/signal transducer and activator of transcription 3 signaling [189,190]. Nevertheless, the observation that fast-twitch muscle is especially reliant upon autophagy to maintain its health highlights important considerations in circumstances where glycolytic fibers are preferentially recruited, such as during eccentric loading [191].

In addition to basal autophagy, animal investigations indicate that the autophagy system plays a crucial role in recovering severely injured skeletal muscle [26,192,193]. Nichenko et al. [192] showed that suppressing autophagy significantly impeded the recovery of mitochondrial enzyme activity and muscle strength in mice exposed to localized cardio-toxin (Ctx) injury. This does not seem to be due to global autophagy repression as similar results have been reported in muscle-specific ULK1-KO mice [26]. Interestingly, ULK1-KO does not impede basal skeletal muscle health in young muscle [26], but does impair mitochondrial homeostasis and skeletal muscle contractility with advancing age [194]. Therefore, it seems ULK1-mediated autophagy facilitates skeletal muscle health and recovery during aging and acute skeletal muscle trauma in mice.

ATG16L-KO, which impedes, but does not entirely suppress autophagosome formation, also significantly delays muscle recovery from Ctx injury [195], with these mice exhibiting elevated sarcolemmal damage, smaller regenerating fibers and lower amounts of both proliferating and differentiating satellite cells compared to their autophagy-sufficient littermates. The effect on satellite cells seems to be of particular importance given that autophagy is upregulated during myoblast proliferation and differentiation *in vitro* [196–198]. Furthermore, loss of basal autophagy through satellite cell-specific ATG7-KO reduces the satellite cell pool in young mice, indicating that autophagy can prevent myogenic cell senescence/death [199]. Accordingly, it is believed that autophagy preserves satellite cell proteostasis and provides the necessary energy for mitosis [198,200]. Intriguingly, *myogenin-Cre* ablation of ULK1, which attenuates autophagy in postmitotic myofibers, represses the myogenic program during recovery from freeze injury, suggesting that autophagy within adult myofibers can regulate satellite cell dynamics [193]. Overall, these data show that autophagy facilitates rodent skeletal muscle repair/regeneration through both intrinsic and extrinsic (satellite cell) mechanisms and that these pathways are interrelated.

It is important to note that an increase in autophagy protein content may not translate to a relative increase in autophagic flux. Investigations conducted by Jarrod Call’s laboratory have shown that while autophagy protein content is elevated in mouse skeletal muscle recovering from traumatic freeze injury, 2-photon microscopy analyses show a reduced clearance of autophagosome bound LC3, indicative of attenuated autophagosome clearance [193,201]. The authors postulated that this could be due to an autophagy ‘bottleneck’ whereby accumulating damaged components are not effectively degraded despite increases in autophagosome production [202]. One possible explanation for this may be that lysosomal biogenesis fails to proportionally increase with damage-induced autophagosome formation. For example, mouse cardiac muscle recovering from ischemia reperfusion (I/R) undergoes a robust increase in autophagosome formation yet is accompanied by a decline in the lysosomal marker lysosome-associated membrane protein 2 (LAMP2) [203], while restored expression of LAMP2 significantly improved the clearance of I/R induced autophagosomes [203]. Similar effects have been shown during TFEB overexpression in cultured I/R injured mouse cardiomyocytes [204], suggesting that a general increase in lysosomal number can overcome the ‘bottleneck’. Overall, these data highlight the lysosomal system as a target to improve autophagic flux and subsequently skeletal muscle recovery.

### Autophagy is upregulated in rodent and cellular models of exercise-induced muscle damage

Salminen and Vihko’s seminal work provided that the first indication that autophagy may be upregulated following EIMD [205], reporting that 9 hours of running causes significant myofiber necrosis and inflammatory cell infiltration in mouse quadriceps muscle. Surviving myofibers exhibited mitochondria-containing autophagic-like vacuoles at days 2 and 7 post-injury, indicative of increased autophagosome production. Using a similar model, Salminen and Kihlström [206] observed markers of lysosomal activity increased stepwise with greater dosages of exercise and subsequent tissue injury, suggesting that a more potent autophagic response occurs with higher magnitudes of EIMD. In more recent years, a growing body of research has identified a notable relationship between markers of autophagy and rodent mitochondrial dysfunction following EIMD [207–209]. Shang et al. [209] reported that 90 minutes of downhill running increased the LC3-II/LC3-I ratio and co-localization of PTEN-induced putative protein kinase 1 (PINK)/Parkin with dysfunctional mitochondria, for up to 48 hours post-exercise. The accumulation of PINK1 and its E3-ligase effector Parkin on depolarized mitochondrial membranes are key stages of ubiquitin-dependent mitophagy and are often preceded by mitochondrial fission events (see Erlich and Hood [210]). Mechanistically, it has recently been reported that the high mobility group box-1 protein (HMGB1), a structural component of chromatin [211], promotes autophagy induction during recovery from downhill running by translocating to the cytosol and relieving BCL-2’s inhibition of Beclin-1 (Figure 3) [207]. A similar mechanism is noted in murine fibroblasts where mitochondrial ROS promote HMGB1-translocation and Beclin-1 mediated autophagy, whereas HMGB1 ablation impedes autophagy, resulting in apoptosis [212]. As such, HMGB1-translocation seems to be a key autophagic signal during conditions that stimulate mitochondrial stress (e.g. EIMD).

Maintaining a healthy mitochondrial pool is crucial for myofiber viability, especially during periods where intracellular calcium homeostasis is perturbed [213]. Investigations of mice with skeletal muscle dystrophy, where calcium [214] and autophagic [215] dysfunction are exhibited, illustrate that an inability to sequester damaged mitochondria exacerbates muscle degeneration and apoptosis during recovery from acute endurance exercise (EE) [216,217]. Mitochondrial turnover is likely important for general skeletal muscle recovery, given the significant energy cost of MPS [218,219]. Indeed, alike autophagy suppression [195,197], inhibition of mitochondrial biogenesis attenuates muscle regeneration following traumatic injury [220]. While the relationship between mitophagy and myogenesis is yet to be explored in the context of EIMD, mitochondrial oxidative stress peaks 6 hours after strenuous EE and coincides with elevated markers of mitophagy, suggesting that mitochondrial turnover and ROS-emitted by sustained oxidative phosphorylation are coupled during exercise recovery [99]. For an in-depth discussion of how mitophagy and mitochondrial turnover may regulate cellular bioenergetics for muscle remodeling, readers are referred to other recent publications [221,222].

It is important to note that the combined aerobic and mechanical stimulus induced by running-based models of EIMD make it difficult to determine whether autophagy is specifically induced to accommodate mechanically induced damage. In fact, many studies have confirmed that autophagic flux is upregulated in rodents following exercise without significant eccentric strain, *i.e*. uphill running and swimming [223–225], which is perhaps unsurprising considering autophagy’s role in mitochondrial remodeling [226]. However, there is some evidence to suggest that eccentric loading promotes a unique autophagic response. Inducible ATG7-KO female mice display impaired exercise capacity compared to their autophagy-sufficient wild-type littermates during downhill, but not uphill, running [208], although limited morphological alterations were observed. In contrast, Lu et al. [227] showed that an unaccustomed bout of exhaustive wheel running induced immediate myofibrillar damage and elevated markers of chaperone-assisted selective autophagy (CASA) (see Tedesco et al. [228] for an in-depth CASA review). Considering skeletal muscle is more susceptible to damage from eccentric loading, an attractive hypothesis may be that resistance-type exercise induces a particularly potent autophagic response. However, in anesthetized rodents, electrically evoked eccentric, concentric, and isometric loading patterns all increase the phosphorylation of ULK1^ser757^ and ULK1^ser317^ to a similar degree without affecting LC3-II/LC3-I ratios [229]. Therefore, further investigations are warranted to determine whether autophagy flux is specifically increased in response to voluntary eccentric-EIMD.

### Autophagy may be upregulated in untrained human skeletal muscle recovering from novel resistance exercise

It is well known that RE, especially involving novel eccentric contractions [4,142], damages myofiber structure [6,136–139] and promotes a robust protein synthetic response [10,11,230]. In untrained individuals, RE-induced myofibrillar damage and protein synthesis are closely related, supporting the notion that muscle proteins are initially synthesized to renew damaged myofibers [5,6,231]. Despite this, human studies investigating the effect of unaccustomed RE on autophagy are equivocal (Table 1) [232–238].

**Table 1:** Overview of human studies investigating effects of acute novel resistance exercise on skeletal muscle autophagy markers in healthy untrained individuals.

Study	Participants	Exercise/Intervention	mRNA expression	Protein content
Diaz-Castro et al. (2024) (232)	Untrained adult males *n* = 8 Age 31.5 ± 4.23 y Weight 78.45 ± 6.29 kg	Unilateral leg presses 10 sets of 10 repetitions at 70% 1RM with 2 minute rest intervals	N/A	(vs. rested leg) LC3AB-II + 1 h↔ LC3AB-II/LC3AB-I + 1 h↔ p62 + 1 h↔ p-AMPK^thr172^ + 1 h↔ p-mTOR^ser448^ + 1 h↑ PINK1 + 1 h↔ Parkin + 1 h↓ BNIP3L/NIX + 1 h↓ FUNDC1 + 1 h↔ BNIP3 + 1 h↓†^2^ MFN2 + 1 h↓†^2^ OPA1 + 1 h↔ p-DRP1^ser616^ + 1 h↑
Dickinson et al. (2017) (238)	Untrained older males control group *n* = 7 Age 74 ± 2 y BMI 26 ± 1 Leucine group *n* = 8 Age 71 ± 3 y BMI 27 ± 1	Bilateral leg extensions 8 sets of 10 repetitions at 65% 1RM with 3-minute rest intervals 10g EEA beverages containing either 1.85 g leucine (control group) or 3.5 g leucine (leucine group) consumed 1 hour post exercise	Control group (vs. baseline) LC3 + 2 h↑ + 5 h↑ + 24 h↑ GABARAP + 2 h↔ + 5 h↔ + 24 h↔ BECN1 + 2 h↔ + 5 h↔ + 24 h↔ ATG7 + 2 h↔ + 5 h↔ + 24 h↔ LAMP2B + 2 h↔ + 5 h↔ + 24 h↔ CISD2 + 2 h↔ + 5 h↔ + 24 h↔ RUNX1 + 2 h↔ + 5 h↑ + 24 h↑ MuRF1 + 2 h↑ + 5 h↑ + 24 h↔ Leucine group (vs. baseline) LC3 + 2 h↑ + 5 h↔ + 24 h↔ GABARAP + 2 h↔ + 5 h↔ + 24 h↔ BECN1 + 2 h↔ + 5 h↔ + 24 h↔ ATG7 + 2 h↔ + 5 h↔ + 24 h↔ LAMP2B + 2 h↔ + 5 h↔ + 24 h↔ CISD2 + 2 h↔ + 5 h↔ + 24 h↑ RUNX1 + 2 h↑† + 5 h↑ + 24 h↑ MuRF1 + 2 h↑ + 5 h↑ + 24 h↔	Control group (vs. baseline) LC3B-I + 2h↔+ 5h↔+24 h↔ LC3B-II + 2 h↓ + 5 h↔*^1^ + 24 h↔ LC3B-II/LC3B-I + 2 h↓ + 5 h↓*^1^ + 24 h↓*^1^ Beclin-1 + 2 h↔ + 5 h↔ + 24 h↔ p-AKT^thr308^ + 2 h↑†^2^ + 5 h↔ + 24 h↔ Nuclear/cytosolic FoxO3a + 2 h↔ + 5 h↔ + 24 h↔*^1^ Leucine group (vs. baseline) LC3B-I + 2 h↔ + 5 h↔ + 24 h↔ LC3B-II + 2 h↓ + 5 h↔*^1^ + 24 h↔ LC3B-II/LC3B-I + 2 hr↓ + 5 h↓*^1^ + 24 h↔*Beclin-1^1^ + 2 h↔ + 5 h↔ + 24 h↔ p-AKT^thr308^ + 2 h↑† + 5 h↔ + 24 h↔ Nuclear/cytosolic FoxO3a + 2 h↔ + 5 h↔ + 24 h↔*^1^
Fry et al. (2013) (233)	Untrained younger adults *n* = 16 Age 27 ± 2 y BMI 25.1 ± 0.9 Untrained older adults *n* = 16 Age 70 ± 2 y BMI 24.2 ± 0.6	Bilateral leg extensions 8 sets of 10 repetitions at 70%1RM with 3 minute rest intervals	Younger group (vs. baseline) LC3 + 3 h↔ + 6 h↔ + 24 h↔ GABARAP + 3 h↓ + 6 h↓†^2^ + 24 h↔ MuRF1 + 3 h↑ + 6 h↑ + 24 h↔ Older group (vs. baseline) LC3+ 3 h↔ + 6 h↔ + 24 h↔ GABARAP + 3 h↓ + 6 h↓†^2^ + 24 h↔ MuRF1 + 3 h↑ + 6 h↑ + 24 h↔	Younger group (vs. baseline) LC3B-I + 3 h↔ + 6 h↔ + 24 h↔ LC3B-II + 3 h↔ + 6 h↓ + 24 h↓ LC3B-II/LC3B-I + 3 h↓ + 6 h↓ + 24 h↓ Beclin-1 + 3 h↔*^1^ + 6 h↔* ^1^+ 24 h↔*^1^ ATG7 + 3 h↔*^1^ + 6 h↔*^1^ + 24 h↑*^1^ FoxO3a^ser253^ + 3 h↓ + 6 h↓ + 24 h↓ p-AKT^thr308^ + 3 h↑ + 6 h↔ + 24 h↔ Older group (vs. baseline) LC3B-I + 3 h↔ + 6 h↔ + 24 h↔ LC3B-II + 3 h↓ + 6 h↓ + 24 h↓ LC3B-II/LC3B-I + 3 h↓ + 6 h↓ + 24 h↓ Beclin-1 + 3 h↔*^1^ + 6 h↔*^1^ + 24 h↔*^1^ ATG7 + 3 h↔*^1^ + 6 h↔*^1^ + 24 h↔*^1^ FoxO3a^ser253^ + 3 h↓ + 6 h↓ + 24 h↓ p-AKT^thr308^ + 3 h↑ + 6 h↔ + 24 h↔
Glynn et al.(2010) (234)	Untrained adult males EAA + LCHO group *n* = 7 Age 30 ± 2 y BMI 26 ± 1 EAA + HCHO group *n* = 32 default 1 Age 32 ± 1 y BMI 27 ± 1	Bilateral leg extensions 10 sets of 10 repetitions at 70%1RM with 3 minute rest intervals ~20 g EAA beverage containing 0.35 g/kgLM EAA with either 0.5 g/kgLM carbohydrate (EAA + LCHO group) or 1.40 g/kgLM (EAA + HCHO group) consumed straight after 1 hour biopsy time point	EAA + LCHO group (vs. baseline) MuRF1 + 1 h↑ + 2 h↑ EAA + HCHO group (vs. baseline) MuRF1 + 1 h↑ + 2 h↑	EAA + LCHO group (vs. baseline) LC3B-I + 1 h↔ + 2 h↔ LC3B-II + 1 h↔ + 2 h↓ p-AMPK^thr172^ + 1 h↑ + 2 h↑*^1^ p-AKT^ser473^ + 1 h↑ + 2 h↑ FoxO3a^ser253^ + 1 h↔ + 2 h↔ FoxO3a^ser318/321^ + 1 h↔ + 1 h↔ MuRF1 + 1 h↑ + 2 h↔ EAA + HCHO group (vs. baseline) LC3B-I + 1 h↔ + 2 h↔ LC3B-II + 1 h↔ + 2 h↓ p-AMPK^thr172^ + 1 h↑ + 2 h↔*^1^ p-AKT^ser473^ + 1 h↑ + 2 h↑ FoxO3a^ser253^ + 1 h↔ + 2 h↔ FoxO3a^ser318/321^ + 1 h↔ + 1 h↔ MuRF1 + 1 h↑ + 2 h↔
Hentilä et al.(2018) (235)	Untrained younger adult males *n* = 12 (whey protein group *n* = 6, placebo group *n* = 6) Age 27 ± 4 y Untrained older adults *n* = 8 Age 61 ± 6 y BMI 23.4–28.8	Bilateral leg presses 5 sets of 10 repetition maximums with 2 minute rest intervals Young cohort consumed either 15 g whey protein isolate or an isocaloric placebo immediately before and after the resistance exercise bout (pooled analysis, no effect of supplementation on autophagy markers)	Younger group (vs. baseline) LC3B + 1 h↔ + 48 h↔p62 + 1 h↔ + 48 h↔	Younger group (vs. baseline) LC3B-I + 1 h↔ + 48 h↑ LC3B-II + 1 h↓ + 48 h↑p62 + 1 h↔ + 48 h↑ p-ULK1^ser555^ + 1 h↓ + 48 h↔ p-ULK1^ser757^ + 1 h↑† + 48 h↔ Beclin-1 + 1 h + 48 h↑†^2^ BCL-2 + 1 h↔ + 48 h↔ Older group (vs. baseline) LC3B-I + 48 h↔ LC3B-II + 48 h↔ p62 + 48 h↔ Beclin-1 + 48 h↔ BCL-2 + 48 h↔
Mazo et al.(2021) (236)	Untrained adult males *n* = 6 Age 27 ± 3 y Weight 79 ± 10 kg	Unilateral leg extensions 8 sets of 10 repetitions at 60-65%1RM with 3 minute rest intervals 40 minutes of stationary cycling at 75% peak heart rate Cross-over trial comparing an unaccustomed bout of aerobic and resistance exercise, separated by ~ 1 week	Resistance exercise (vs. baseline) Autophagy genes + 1 h 7 ATGs DE + 4 h 43 ATGsDE FOXO3 pathway + 1 h↑ + 4 h↑ mTOR pathway + 1 h? + 4 h↑ Aerobic exercise (vs. baseline) Autophagy genes + 1 h 6 ATGs DE + 4 h 17 ATGsDE FOXO3 pathway + 1 h↑ + 4 h? mTOR pathway + 1 h? + 4 h↑	Resistance exercise (vs. baseline) LC3B-I + 1 h↔ + 4 h↔ LC3B-II + 1 h↓ + 4 h↓ LC3B-II/LC3B-I + 1 h↔ + 4 h↔ p62 + 1 h↔ + 4 h↓ FoxO3a + 1 h↔ + 4 h↓ mTOR^ser2448^ + 1 h↔ + 4 h↑*^1^ Aerobic exercise (vs. baseline) LC3B-I + 1 h↔ + 4 h↔ LC3B-II + 1 h↓ + 4 h↓ LC3B-II/LC3B-I + 1 h↔ + 4 h↔ P62 + 1 h↔ + 4 h↓ FoxO3a + 1 h↔ + 4 h↓ mTOR^ser2448^ + 1 h↔ + 4 h↑†*^2^,^1^
Ogborn et al. (2015) (237)	Untrained younger males *n* = 9 Age 21 ± 3 y Weight 91.7 ± 21.9 kg Untrained older males *n* = 9 Age 70 ± 4 Weight 87.6 ± 11.5 kg	Unilateral leg extensions and leg presses 4 sets of 10 repetitions per exercise at 75%1RM with 2-minute rest intervals (pooled analysis, no effect of age)	(vs. rested leg) LC3B + 3 h↑ + 24 h↑†^2^ + 48 h↔ p62 + 3 h↑ + 24 h↔ + 48 h↔ ATG7 + 3 h↔ + 24 h↔ + 48 h↔ BECN1 + 3 h↔ + 24 h↔ + 48 h↔ VPS34 + 3 h↔ + 24 h↔ + 48 h↔ BNIP3 +3h↔+24h↔ + 48 h↔	(vs. rested leg) Total LC3B + 3 h↔ + 24 h↑†^2^ + 48 h↑ p62 + 3 h↔ + 24 h↑ + 48 h↑ ATG7 + 3 h↔ + 24 h↔ + 48 h↔ PINK1 + 3 h↔ + 24 h↔+ 48 h↔ Parkin + 3 h↔ + 24 h↔ + 48 h↔

1 Denotes a significant difference between condition/group.

2 Indicates a borderline significant difference compared to baseline or rested sample.

One related hypothesis on this topic is that mechanical stimulation of AKT/mTORC1 activity inhibits autophagy following acute RE [18,235,239]. In support of this, Hentilä et al. [235] showed that LC3-II, but not LC3-I or p62, protein content decreased 1 hour post-exercise, in line with reductions in AMPK-dependent ULK1^ser555^ and elevations in mTORC1-dependent ULK1^ser757^ phosphorylation. Several autophagy-related proteins were, however, elevated once ULK1^ser757^ returned to basal values 48 hours post exercise, which could suggest that autophagosome formation increases once anabolic signaling has diminished. Alternatively, it is possible that autophagy proteins may have accumulated due to inhibited autophagosome degradation during recovery, thereby corroborating the ‘bottleneck’ hypothesis [202]. Regulation of autophagy-related genes following novel RE is also equivocal, with studies reporting unaltered [233,235] or elevated [237] LC3 and p62 expression. The combination of novel RE and essential amino acid (EAA) ingestion, a further anabolic stimulus [8], elicits reductions in LC3-II/LC3-I ratio at 2 h post-exercise, possibly reflecting a reduction in autophagosome biogenesis. Interestingly, if the EAA beverage contained higher leucine doses, the LC3-II/LC3-I ratio increased at 5 and 24 hours, leading the authors to postulate that further elevations in mTORC1 activation may have impeded autophagosome degradation [238,240]. The observation that LC3-II increases with higher amounts of leucine ingestion is in contrast to *in vitro* [241] and animal investigations [242], which characterize leucine-mediated autophagy inhibition through a reduction in LC3-II content. However, as each of these studies only utilized ‘static’ assessments of autophagic protein content, more appropriate methodologies are required to better understand the relationship between RE, nutritional status, and autophagy flux.

Alike novel RE, Fritzen et al. [243] showed that 1 h one-legged concentric cycling exercise (80% peak workload with two 5-minute intervals at 100%) reduced LC3-II/LC3-I ratio, but not p62 content, for up to 4 hours post-exercise in moderately trained men. This occurred alongside elevated AMPK-dependent ULK1^ser555^ phosphorylation, implying that while EE upregulates AMPK/ULK1 signaling, this may not lead to altered autophagosome content [243], or could simply reflect that static measurements are unable to accurately reflect autophagic flux. Other works have reported that exercise-induced ULK1^ser555^ phosphorylation (AMPK-regulated), but not ULK1^ser757^ (mTORC1), was associated with a lowered LC3-II/LC3-I ratio immediately after 1 h cycling at 50% maximal oxygen consumption (VO_2max_), potentially indicating elevated autophagosome clearance [244]. p62 protein content was, however, unaltered in this investigation, highlighting further uncertainty over whether autophagy flux was increased. In contrast, Mazo et al. [236] showed that acute cycling (40 minutes at 75% peak-heart rate) or lower-body RE reduced both LC3-II and p62 protein content to a similar degree in untrained individuals 4 hours after exercise. Considering that both LC3-II and p62 are degraded within the lysosomal lumen during autophagy [77], autophagosome degradation may have been upregulated, a notion reinforced by the observation that autophagy gene expression was upregulated at 1 h and 4 hours post-exercise. Nevertheless, while combined measures of p62, LC3-II, and upstream autophagic regulators provide a more detailed description of autophagy, these findings cannot confirm the status of autophagic flux, which requires the use of autophagosome-lysosome fusion inhibitors [77].

Despite the evident limitations of monitoring autophagy via static measures, Schwalm et al. [245] found that the LC3-II/LC3-I ratio and p62 protein content was decreased in endurance-trained individuals 1 hour after high-intensity (70% VO_2max_), but not low-intensity (55% VO_2max_) cycling. In addition, autophagy-related gene expression was greater in the high-intensity trial and these effects were primarily attributed to a greater induction of the AMPK/ULK1 axis, possibly indicating elevated autophagy induction and autophagosome clearance with higher intensity exercise. However, as the total amount of work performed differed between trials, it is unclear whether the decline in autophagy proteins was specifically related to exercise intensity. For example, work-matched bouts of exercise performed above, or below, maximum lactate steady state elicit similar reductions in LC3-II protein content albeit while p62 protein content and autophagy-related gene expression were unchanged [246]. These data may indicate that total work completed during an exercise bout may be the primary driver of alterations in autophagy, although further work utilizing more sophisticated measures of autophagic flux in human skeletal muscle is required to confirm this.

In addition to EE, RT can induce mitochondrial remodeling [247–251]; thus, it is plausible that auto/mitophagy pathways may be regulated by this form of exercise. Diaz-Castro et al. [232] recently reported that acute RE increased markers of mitochondrial fission and elicited reductions in the protein content of the mitophagy receptor BNIP3L/NIX in untrained human muscle. TEM-derived observations of damaged mitochondria and mitophagosome-like structures were also noted at this time, suggesting that mitophagy may have been taking place. It is unclear whether the mitophagosome-like structures were degraded intracellularly as LC3-II and p62 content were unchanged. Intriguingly, though, it was also reported that subsarcolemmal mitophagosome-like structures could be seen exiting muscle following RE, a phenomenon that has been described in other cell types [252,253]. While Diaz-Castro et al. [232] did not show evidence of mitophagosomes in circulation, other studies have shown that mouse cardiac muscle with impaired lysosomal function also ejects damaged mitochondria, but the vesicles do not appear in circulation due to being degraded by nearby macrophages [254]. Similar ‘outsourcing’ of mito-/autophagy has also been observed within human mesenchymal stem cells [255], and pharmaceutical inhibition of lysosomal function elevates secretory autophagy [256,257]. Considering these data, extracellular release of autophagosome-like structures could represent an alternative mechanism to eliminate cellular debris when lysosomes are inundated during recovery from novel RE (i.e. the ‘bottleneck’) [232], although further research regarding this novel hypothesis is required.

## Limitations of this field and outstanding questions

### Static measures of autophagy are insufficient to determine autophagic flux

As emphasized throughout this review, a key limitation of human research is the use of static markers to infer the status of autophagy flux. Cellular contents of LC3, p62, and many other autophagy-related proteins change depending on their rate of synthesis and/or their autophagic degradation, meaning that unidirectional changes could be due to either elevated autophagosome formation or reduced fusion with lysosomes and subsequent degradation. Drugs that block autophagosome degradation (e.g. colchicine and bafilomycin) have proven invaluable in determining the autophagic effect of exercise in animals and cellular models of muscle contraction [77]; however, the use of these drugs in human research is ethically challenging. By adapting such an assay [258], Botella et al. [246] have recently provided some, albeit limited, evidence that EE may upregulate autophagy flux in human skeletal muscle. In agreement with most investigations, static measures of human LC3-II protein content were immediately reduced post-exercise and returned to basal values within 3.5 hours of recovery, with no changes in p62 protein content or related mRNA expression. In contrast, in rodents, LC3-II was unaffected immediately after but increased 3.5 hours post-exercise, corroborating most reports that static measures of LC3-II decrease in human [233–236,238,243–245] and increase in rodent [17,110,111,208,217,227,259–261] skeletal muscle during the initial stages of exercise recovery. However, when a small subset of biopsied tissue (*n* = 5) was incubated in a lysosomotropic ammonium chloride-leupeptin solution prior to freezing, a moderate-to-large effect of exercise on elevating LC3-II flux was observed for up to 24 hours [246]. These data should be interpreted with caution given the limited sample size, but they do indicate the potential for this methodology to be utilized in human exercise studies.

**Figure 4:**
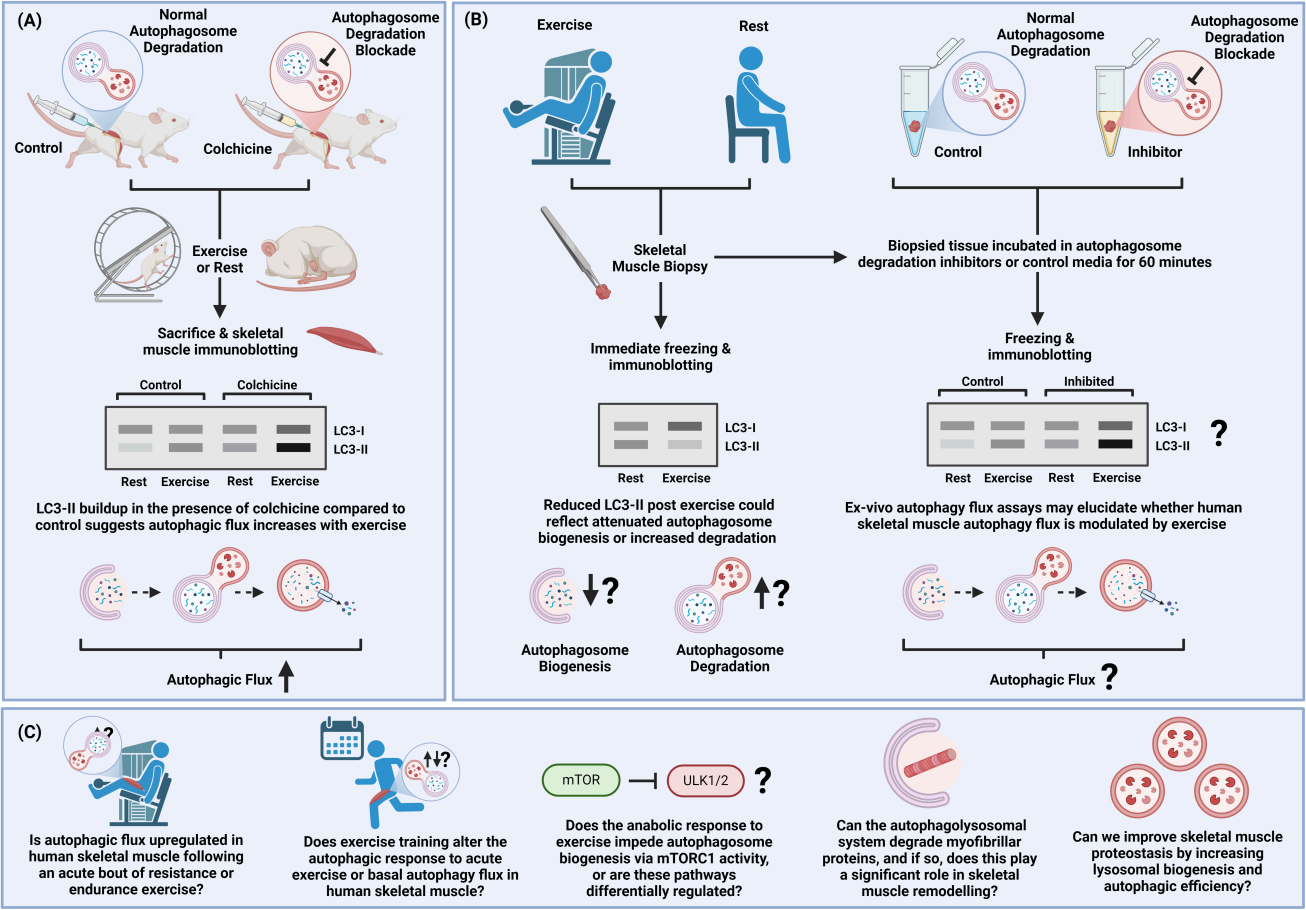
Limitations of current research and future questions. (**A**) Experimental model commonly used to measure autophagic flux in rodents. (**B**) Schematic of how an *ex vivo*autophagy flux methodology could be incorporated into existing human muscle biopsy techniques to better infer activity of the autophagolysosomal system. (**C**) A list of pertinent questions yet to be elucidated by researchers studying human skeletal muscle autophagy.

Overall, comparing human and rodent changes in static measures of LC3 may lead to false conclusions regarding the autophagic response to exercise. Although a confirmatory lysosomal blockade can delineate whether LC3-II is altered by lysosomal degradation, LC3-II is also a feature of phagocytic and endocytic pathways that converge at the lysosome [262]. Therefore, the flux of other autophagy-related proteins could also be included to provide a better indication of whether the autophagolysosomal system is specifically upregulated. We hope that future experiments employing *ex vivo* autophagy assays will shed light on conflicting data and ultimately begin to decipher the complexity of autophagy regulation in human skeletal muscle (Figure 4).

### Potential mTOR-independent mechanisms of selective-autophagy at sites of cellular damage

A primary reason why many researchers postulate that RE could lead to inhibition of autophagy is due to mTORC1’s inhibitory effects on aspects of the autophagic cellular machinery [78,86]. However, it is becoming increasingly evident that autophagic pathways can be differentially regulated [263,264], and several accounts of mTORC1-independent autophagic induction have been described in *in vitro* and animal studies. For example, Cardenas et al. [265,266] identified an AMPK-mediated mechanism of autophagy activation in response to alterations in ER-mitochondria calcium flux, which occurs regardless of mTORC1 activity status. Moreover, others have displayed ULK1-mediated autophagy to occur at mitochondria and peroxisomes independent of both AMPK and mTORC1 [267] and investigations in C2C12 myotubes show that CASA-mediated degradation of filamin can occur despite mechanically induced mTORC1 activation [268]. In addition to autophagy induction events, transcriptional regulation of the autophagolysosomal system can occur independent of mTORC1. Medina et al. [108] reported that local calcium release from the lysosomal calcium channel MCOL1N activates nearby calcineurin, which, in turn, dephosphorylates TFEB^Ser211/Ser142^ allowing its nuclear translocation even in nutrient-replete conditions. The precise mechanism governing MCOL1N activation in exercised skeletal muscle is unclear, although mitochondrial-ROS have been shown to stimulate the MCOL1N/TFEB axis in COS-I and HEK293 cells [106,107]. It is plausible that such mTORC1-independent mechanisms could occur in human skeletal muscle following RE to elevate autophagic flux, albeit well-controlled experimental investigations are required to elucidate this.

### Does autophagy contribute to myofibrillar protein degradation?

**Figure 5:**
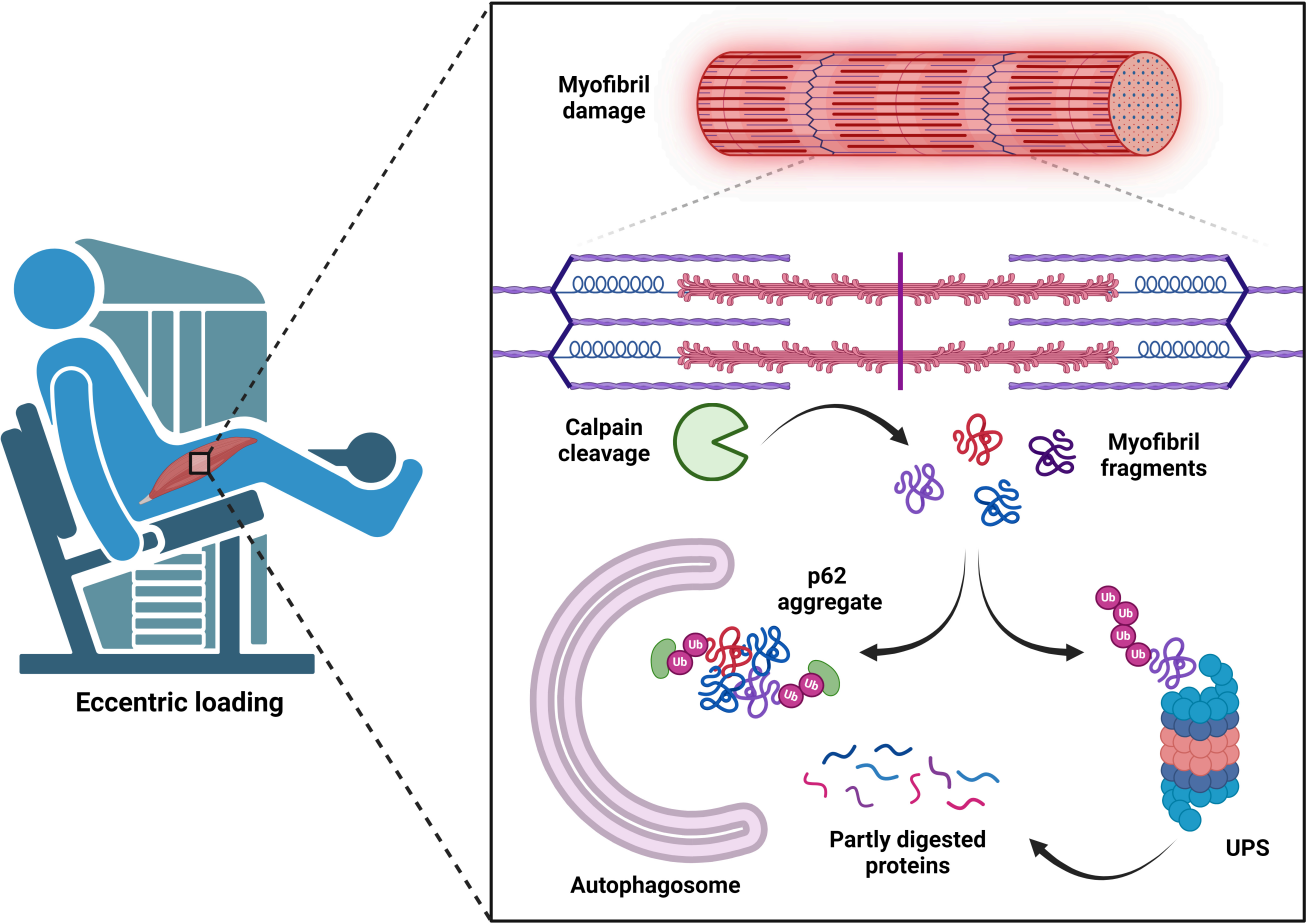
Hypothetical model of contractile protein degradation by the autophagy system. Myofibrillar proteins can become damaged during periods of mechanical loading and must, therefore, be removed for muscle recovery to ensue. The calpains release myofibrillar proteins from sarcomeres allowing the ubiquitinated fragments to be degraded by the UPS. The autophagy system may also contribute to the degradation of aggregated myofibrillar fragments, or partly degraded peptides released from the UPS. Created in BioRender. Acheson, J. (2024) BioRender.com/f57e974.

Another issue often discussed in the field of muscle protein metabolism is whether autophagy can contribute to myofibrillar protein turnover. Early research demonstrated that lysosomal protease inhibitors do not suppress proxy measures of myofibrillar protein breakdown [269], and immunofluorescence imaging of starved rat skeletal muscle shows cytoplasmic autophagosomes with no evidence of enclosed myofibrillar proteins [25]. Therefore, it is generally believed that the proteasomal and calpain systems are primarily responsible for myofibrillar protein turnover while the lysosomal system facilitates degradation of cytosolic proteins and organelles [20,270]. However, *in vitro* investigations have shown that lysosomal cathepsins can hydrolyze purified myofibrillar proteins [271–273] and recent transmission electron microscopy (TEM) images of human and rodent skeletal muscle following EIMD observe autophagosome-like structures within the intermyofibrillar space [127,207,227,274]. Furthermore, autophagic flux is upregulated in aging rodent skeletal muscle with impaired proteasomal function and significant protein aggregation, possibly reflecting compensation of the autophagy system [18]. Interestingly, autophagic flux is not further enhanced in these animals during recovery from disuse atrophy, which could suggest that lysosomes are inundated in basal conditions and cannot cope with an elevated need to degrade accumulating aggregates. Therefore, autophagy may operate in harmony with other proteolytic pathways to sufficiently degrade cleaved contractile proteins or aggregated peptide chains during periods of myofibrillar damage (Figure 5).

One such investigation that has observed autophagy-related myofibrillar degradation in humans is Ulbricht et al. [274], whereby an acute bout of maximal eccentric contractions, but not conventional RE, elicited myofibrillar disruption and reductions in CASA proteins and their substrate, filamin C. Immunofluorescence imaging of damaged fibers showed an increase in LC3-positive structures suggesting autophagosome presence/formation in this region. Furthermore, the mechanical stretch of the myofibrillar protein titin in rat cardiomyocytes exposes a cryptic titin-kinase binding site, which can associate with autophagy adaptors neighbor of BRCA1 gene 1 (NBR1) and p62 [275]. More recent data indicate that muscle inactivity may promote an interaction between NBR1/p62 and titin-kinase at sarcomeres, further indicating a potential role of autophagy in myofibrillar turnover [276]. Nevertheless, further work is required to confirm whether autophagy flux is enhanced during periods where CASA protein content decreases [274] as well as to determine whether this pathway contributes to myofibrillar protein turnover.

### Is autophagy involved in skeletal muscle adaptation to chronic exercise?

The repeated bout effect increases skeletal muscle’s resilience to mechanical damage [4], contributed to by a variety of adaptations including a sensitized immune response, extracellular matrix remodeling, and neural modulation [3]. However, there is some evidence that the autophagy system is also modulated by consistent training. Ulbricht et al. [274] observed protein levels of p62 and several CASA components to be elevated after 4 weeks of progressive RT, but not constant load RT, suggesting that progressive overload is required for adaptation to the autophagolysosomal system. Conversely, others have shown no changes in p62 protein content following 12 weeks of progressive RT [277], although this study was conducted in trained men where adaptations could have already occurred. It is also possible that different types of RE may induce unique adaptations in the autophagy system. Lim et al. [278] reported that 10 weeks of low-load, high-volume RT (30%1RM) performed to volitional fatigue increased Parkin protein content and proteins involved in mitochondrial dynamics, yet these adaptations did not occur in high-load or low-intensity non-failure training cohorts. Thus, high-volume, low-load RT may promote a greater capacity for mitochondrial remodeling, possibly to accommodate mitochondrial stress elicited by a more ‘endurance-type’ exercise. Indeed, 8 weeks of continuous moderate-intensity or work-matched sprint interval endurance training (ET) increases Parkin, BNIP3, LC3-I, and oxidative phosphorylation complex 1 content in previously moderately trained men [279]. Similarly, in mouse skeletal muscle, 6 weeks of ET increased mitochondria content, Parkin expression, and Parkin-colocalization with mitochondrial markers [280], although basal levels of mitophagy flux were unchanged, further highlighting the importance of including measurements of flux in such investigations. Other studies investigating the effects of chronic exercise training on ‘static’ measures of autophagy protein content observe contrasting results with several reporting increased LC3-I content [243,279], which could indicate increased autophagic capacity without alterations in basal autophagosome content, while others suggest that the LC3-II content is increased, indicating potential expansion of the autophagosome pool [235,246].

Another model that has provided insight into the regulation of the autophagolysosomal system to exercise training is that of chronic contractile activity (CCA), employed consistently by the laboratory of Prof. David Hood. Here, several investigations have indicated that frequent bouts of muscle contraction enhance the content of proteins that regulate autophagy induction (e.g. LC3-I, Beclin-1) and related transcriptional programs [100,281–283]. Paradoxically, however, when utilizing autophagy inhibitors, CCA had either no effect or reduced basal LC3-II/p62 flux, potentially reflecting improvements in muscle quality [100,281,283,284]. The acute increase in autophagy flux during EE recovery is also attenuated in trained-mouse quadriceps, indicating increased resilience to exercise-induced stress [281]. Similarly, 9 weeks of progressive weighted climbing exercise reduces markers of autophagy flux (e.g. LC3-II/LC3-I ratio and p62) while increasing those of autophagic and lysosomal capacity in aging rats [285]. *In vitro* data also support these notions as CCA elicits elevations in TFEB protein content and markers of lysosomal content and proteolytic activity [100,282,284]. Notably, evidence of lysosomal biogenesis can occur within as little as 3 days of increased contractile activity and precedes mitochondrial adaptations in these models [282]. In contrast, TFEB content was unaltered in young mouse skeletal muscle following 9 days of CCA *in vivo* [283], although lysosomal biogenesis did occur. Overall, these data show that TFEB activity and lysosome biogenesis are important mechanisms underpinning skeletal muscle plasticity and may contribute to other autophagic adaptations.

It is unclear whether TFEB nuclear translocation and lysosomal biogenesis are enhanced by exercise training in human skeletal muscle, although we have observed elevations in LAMP2 protein content following 8 weeks of progressive RE [286]. Considering the relationship between CCA and lysosomal biogenesis *in vitro* and in rodent skeletal muscle, we speculate that an enhanced lysosomal pool may increase the efficiency of autophagolysosomal recycling [281]. Furthermore, given that lysosomal biogenesis appears to be the rate-limiting factor of the autophagolysosomal system during skeletal muscle repair (i.e. the ‘bottleneck’ [202]), methods to improve lysosomal capacity may benefit untrained individuals susceptible to EIMD. *In vitro* and animal-based studies have reported that nutraceutical compounds such as curcumin [282,283], spermidine [284,285,287], and quercetin [288,289] can promote lysosomal biogenesis and autophagic function, although the use of these in relation to exercise-induced autophagy have yet to be comprehensively investigated in human skeletal muscle. Importantly, some evidence suggests that antioxidant supplementation can also attenuate autophagy [103,208], highlighting the need for further mechanistic investigation of these compounds.

## Conclusion

The autophagolysosomal system maintains skeletal muscle homeostasis throughout the lifespan and during acute stress such as energy imbalance and tissue injury. Changes in intracellular calcium and redox status are key signals regulating autophagy induction and transcriptional programs. Animal models generally indicate that autophagy is upregulated to remove dysfunctional mitochondria and preserve skeletal muscle integrity during recovery from strenuous exercise. However, a lack of sufficient methods to monitor autophagy flux has prevented any robust conclusions regarding whether autophagic degradation is increased by exercise-related stress in human skeletal muscle. It is well established that novel eccentric exercise is particularly damaging to myofibrillar protein architecture; thus, autophagy may play an important role in muscle regeneration and remodeling. Moreover, consistent exercise training can increase the expression of autophagy-related genes and proteins, suggesting potential adaptation of the autophagy system to increased skeletal muscle loading. Nonetheless, further well-controlled human investigations utilizing various autophagic signaling and flux measurements are required to appropriately delineate whether, and if so, how autophagy contributes to exercise-induced muscle repair/remodeling.

## Abbreviations

AKT: protein kinase B/AKT
AMP: adenosine monophosphate
AMPK: 5′ AMP-activated protein kinase
ATG: autophagy-related gene
ATP: adenosine triphosphate
BCL-2: B-cell leukemia/lymphoma 2 protein
BNIP3: BCL2/Adenovirus E1B 19 kDa protein-interacting protein 3
BNIP3L/NIX: BCL2/Adenovirus E1B 19 kDa protein-interacting protein 3-like
CASA: chaperone-assisted selective autophagy
CCA: chronic contractile activity
CLEAR: coordinated lysosomal enhancement and regulation
CaMKKβ: calcium/calmodulin-dependent protein kinase kinase β
Ctx: cardio-toxin
EAA: essential amino acids
EDL: extensor digitorum longus
EE: endurance exercise
EIMD: exercise-induced muscle damage
ER: endoplasmic reticulum
ERK1/2: extracellular signal-regulated kinases
ESCRT: endosomal sorting complexes required for transport
ET: endurance training
FIP200: focal adhesion kinase family interaction protein of 200kD
FOXO3: forkhead box O3
GABARAP: gamma-aminobutyric acid receptor-associated protein
HMGB1: high mobility group box-1 protein
HOPS: homotypic fusion and protein sorting
I/R: ischemia reperfusion
JNK1: c-Jun N-terminal protein kinase 1
KO: knockout
LAMP2: lysosome-associated membrane protein 2
LC3: microtubule-associated protein 1A/1B-light chain 3
MAPK: mitogen-activated protein kinases
MCOLN1: mucolipin-1/TRPML1
MPB: muscle protein breakdown
MPS: muscle protein synthesis
MiT/TFE: microphthalmia/transcription factor E
NBR1: neighbor of BRCA1 gene 1
PE: phosphatidylethanolamine
PI3K-C1: class III phosphatidylinositol 3-kinase complex 1
PINK1: PTEN-induced putative protein kinase 1
PI3P: phosphatidylinositol 3-phosphate
PIS: phosphatidylinositol synthase
PKCθ: protein kinase C theta
RE: resistance exercise
ROS: reactive oxygen species
RT: resistance training
SNARE: soluble N-ethylmaleimide-sensitive factor attachment protein receptors
SR: sarcoplasmic reticulum
TEM: transmission electron microscopy
TFE3: transcription Factor Binding to IGHM Enhancer 3
TFEB: transcription factor EB
ULK1/2: unc51-like kinase 1/2
UPS: ubiquitin-proteasome
VAPs: VAMP-associated proteins
VO_2max_: maximal oxygen consumption
VPS: vacuolar protein sorting
WIPI: WD repeat domain
mTORC1: mammalian target of rapamycin complex 1
p62: sequestosome-1
v-ATPase: vacuolar H^+^-adenosine triphosphatases
